# Clinical Dilemma - Cardiac Memory vs Myocardial Ischemia

**DOI:** 10.7759/cureus.7129

**Published:** 2020-02-28

**Authors:** Rastko Rakočević, Renjit Thomas, Ralph G Oriscello

**Affiliations:** 1 Internal Medicine, University Hospital - Rutgers New Jersey Medical School, Newark, USA; 2 Pulmonary and Critical Care Medicine, University of Southern California, Los Angeles, USA; 3 Cardiology, University Hospital - Rutgers New Jersey Medical School, Newark, USA; 4 Cardiology/Critical Care, Veterans Affairs (VA) East Orange/Rutgers New Jersey Medical School, East Orange, USA

**Keywords:** cardiac memory, twi, pacemaker, t wave inversion, ischemia, critical care, ischemia trial

## Abstract

Cardiac memory (CM) is a commonly unrecognized entity in which electrocardiograph (EKG) changes demonstrate T wave inversions (TWI) that appear consistent with ischemia. Inability to recognize and distinguish CM from actual ischemia can be a burden for both patients and hospitals, leading to unnecessary hospital admission, cardiac testing, and cardiac catheterization. Simple EKG analysis and meticulous interpretation of T-wave axis and morphology can help differentiate between the two. We present a case with such a dilemma, and an overview literature and physiology behind this entity.

## Introduction

In the right constellation of signs and symptoms, T wave inversions (TWI) on electrocardiograph (EKG) may represent ischemic event that requires immediate care. Orientation to details must be emphasized when interpreting EKGs, or some entities could be missed. Cardiac memory (CM) is one of those physiological manifestations that has specific set of clues that can be observed on a simple 12 lead EKG. With this report we aim to demonstrate some of the difficulties a clinician can encounter during everyday evaluation of patients with initial symptom of chest pressure, with emphasis on EKG interpretation and deeper understanding of the physiology of cardiac memory.

## Case presentation

**Figure 1:**
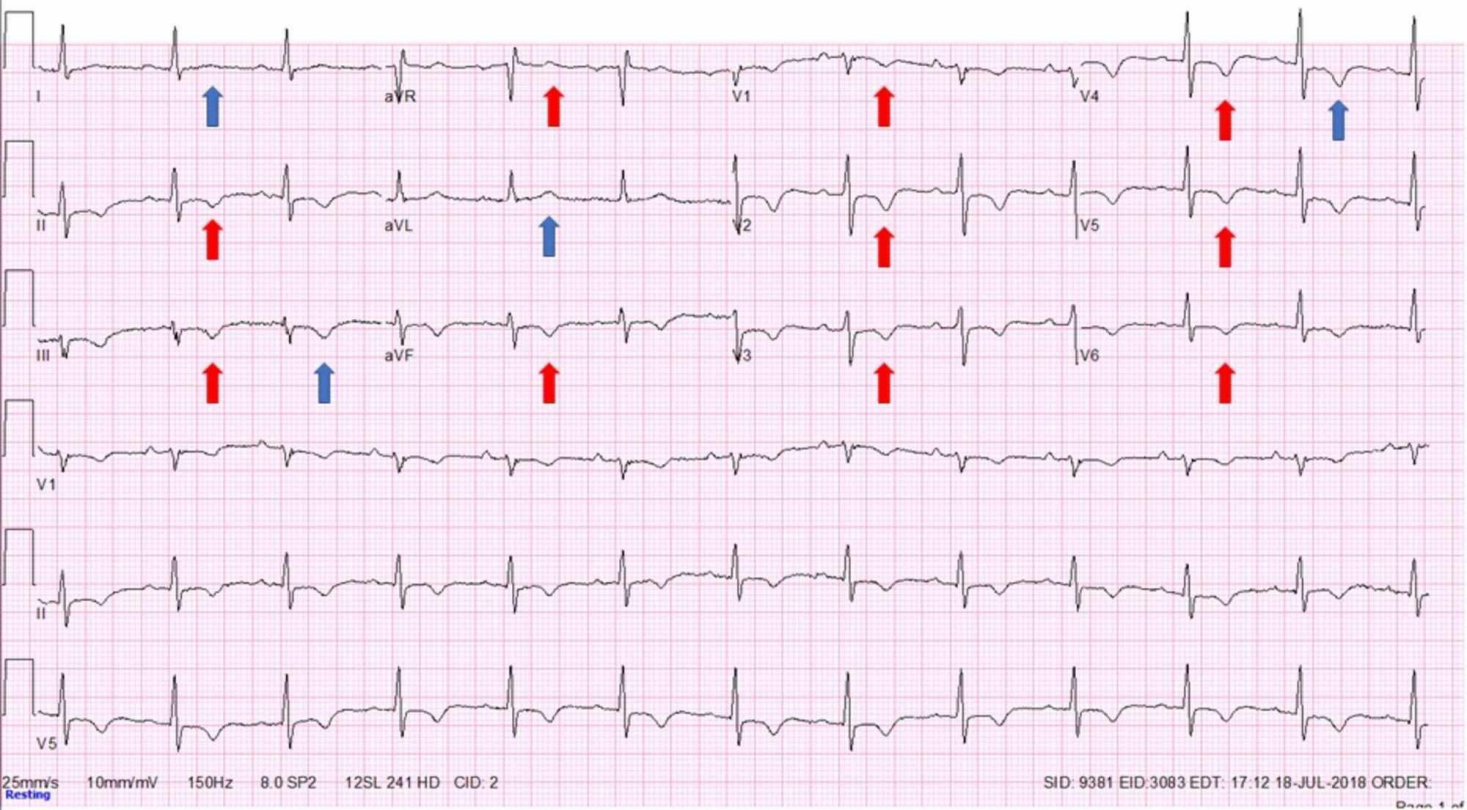
Electrocardiograph (EKG) on initial evaluation. No chest pain. Normal sinus rhythm with diffuse repolarization abnormalities seen as T wave inversions in precordial and inferior leads and positive T wave in aVR

**Figure 2:**
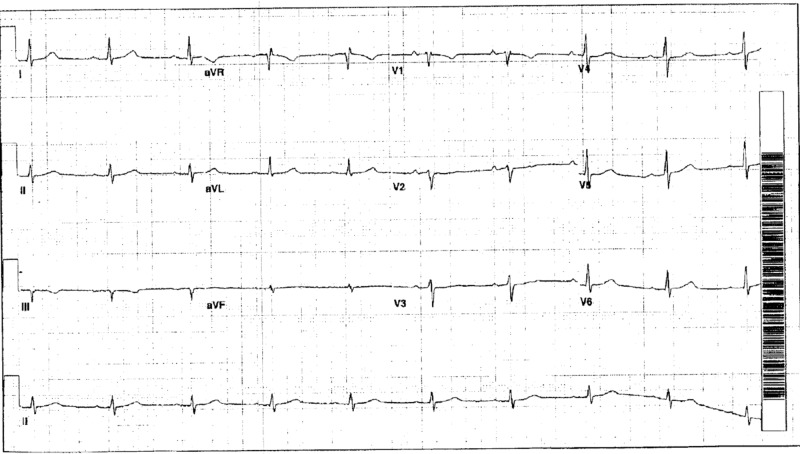
Baseline electrocardiograph (EKG) prior to this presentation. Normal sinus rhythm, no T wave abnormalities

A 54-year-old male army veteran with a past medical history of tobacco and alcohol use disorder (last use eight years ago), current daily marijuana use, post-traumatic stress disorder (PTSD), depression, anxiety, diabetes mellitus, asthma, chronic obstructive pulmonary disease (COPD), obstructive sleep apnea (on home CPAP), factor V Leiden thrombophilia (on warfarin), and paroxysmal atrial fibrillation (pAF, on metoprolol and flecainide) with multiple pAF ablation procedures and sick sinus syndrome with single lead right ventricular pacemaker placement presented for routine evaluation with a chief complaint of PTSD. He was found to have severe shortness of breath on exertion associated with midsternal chest pressure and lightheadedness, that was gradually progressing for the last year. Symptoms occurred after minimal activity (NYHA class III) and were not present at rest. Pressure was sometimes associated with the radiation to his left shoulder and arm. On physical exam he was morbidly obese male, tachypneic with prolonged expiratory phase, irregular heart rhythm, rate of 80 beats per minute, without any murmurs or rubs or any stigmata of heart failure appreciated. Obtained electrocardiographs (EKGs) demonstrated diffuse T wave inversion (TWI) in all the precordial and inferior leads regardless of an underlying rhythm (Figure 1, red arrows), not seen on previous EKGs (Figure 2).

**Figure 3:**
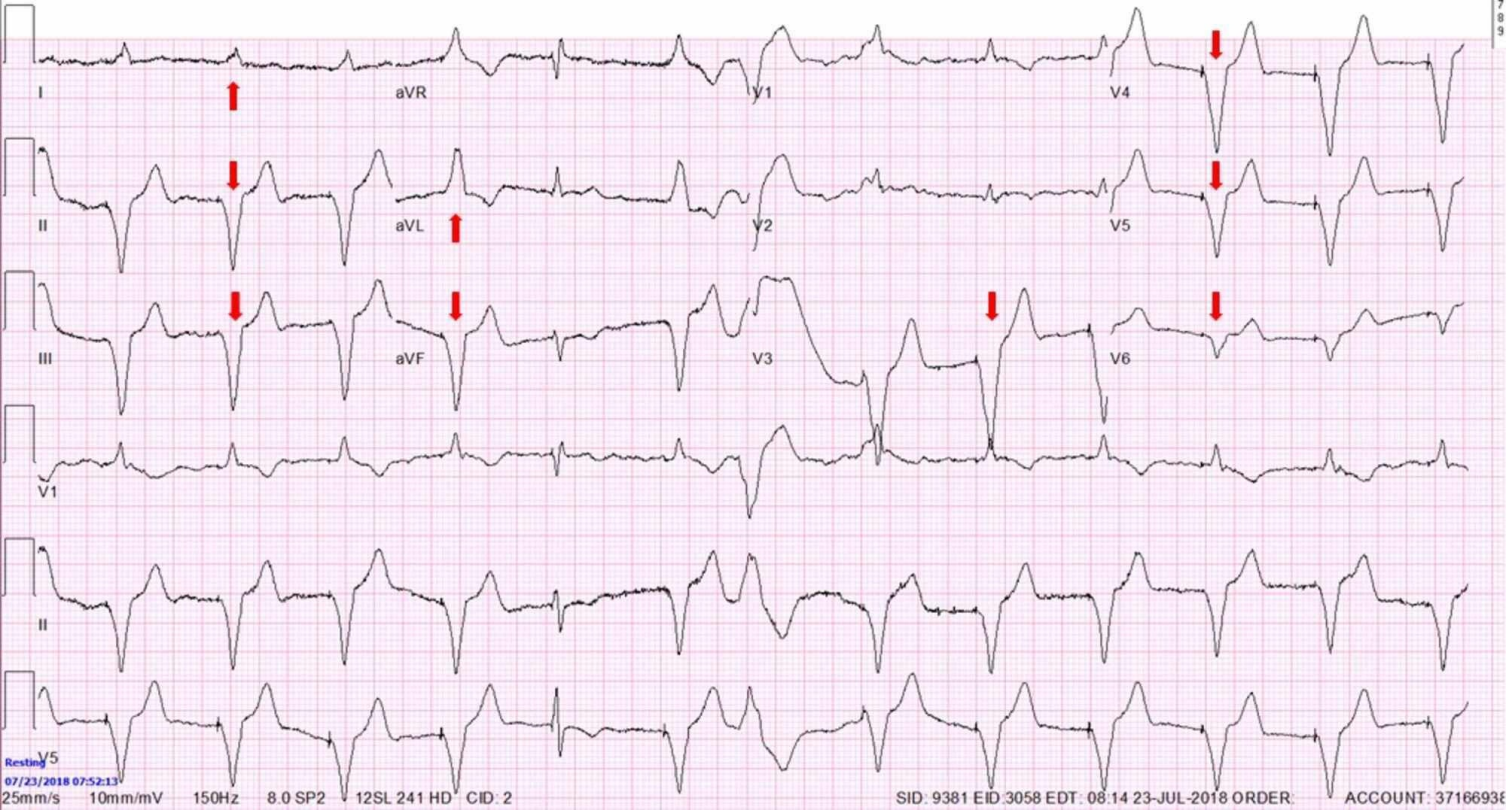
Right ventricular pacing with wide negative QRS complexes in precordial and inferior leads and wide positive QRS complexes in leads I and aVL

Pacemaker device was interrogated with 30% total pacing time and EKG showing typical wide paced QRS complexes (Figure 3, red arrows).

**Figure 4:**
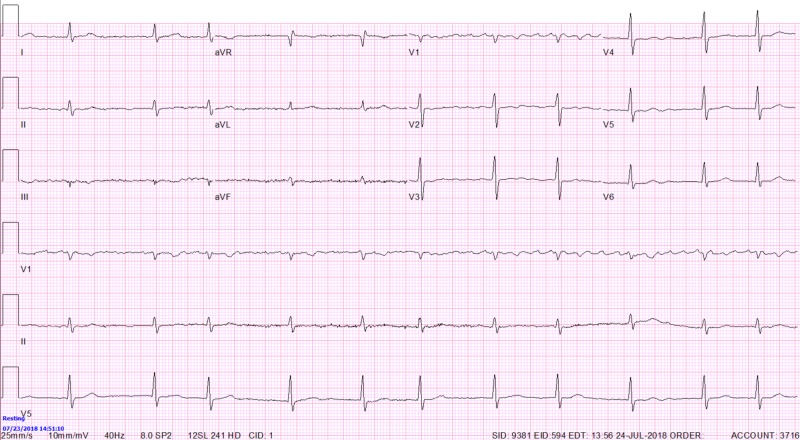
Atrial fibrillation with ventricular rate of 66 with no T wave abnormalities

Nuclear stress test (Lexiscan) demonstrated medium size moderate intensity reversible defect in the apex, anterior, anterolateral, and inferolateral walls extending from the apex to the base. Test also showed left ventricular hypertrophy (LVH) with preserved left ventricular ejection fraction (LVEF) on gated images. Patient was sent for cardiac catheterization which revealed a right dominant vasculature with a severe distal right coronary artery (RCA) and a severe proximal left circumflex artery (LCx) disease with 90% and 80% stenosis, respectively, and TIMI Flow score 3 in both vessels indicating complete flow. There were luminal irregularities in the left anterior descending artery (LAD) and distal LCx. A 2.5 x 33 m Xience drug eluting stent (DES) was deployed in the RCA and post dilated with a 3.0 mm nc balloon and a 4.0 x 18 mm Xience DES was deployed in the LCx and post dilated with a 4.5 mm nc balloon with 0% post procedure stenosis and TIMI score 3 in both vessels. The EKG obtained a day after the procedure demonstrated resolution of TWI (Figure 4).

**Figure 5:**
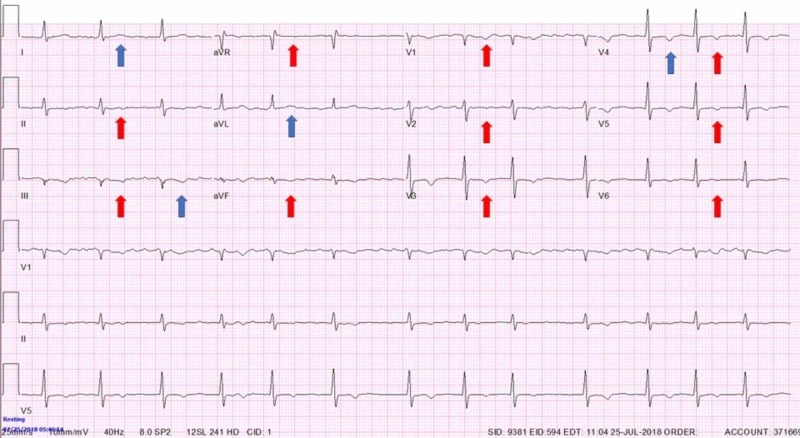
Atrial fibrillation with ventricular rate of around 88 with diffuse repolarization abnormalities seen as T wave inversions in precordial and inferior leads

Overnight, occasional ventricular pacing was observed on the telemetry monitor, with the same morphology seen on previous EKG in Figure 3. The day after, repeated EKG showed again a diffuse TWI in precordial and inferior leads seen before the procedure (Figure 5, red arrows). The patient had no improvement in immediate or remote overall symptoms.

## Discussion

Cardiac memory (CM) is a commonly unrecognized entity in which EKG changes demonstrate TWI that appear consistent with ischemia. Inability to recognize and distinguish CM from actual ischemia can lead to unnecessary hospital admission, cardiac testing, and cardiac catheterization. Analysis of T-wave axis and morphology can help differentiate between the two. CM is persistence of TWI developed after a period of abnormal ventricular activation once normal ventricular activation is restored [1-3]. Abnormal ventricular activation that precedes CM is often due to artificial pacemaker activity but also intrinsic ventricular ectopic focus, like intermittent left bundle branch block, Wolff-Parkinson-White (WPW), ventricular tachycardia [2,4-8]. It was described that the T-wave axis during the CM has the same direction as QRS axis during the abnormal ventricular activation that caused CM (Figure 3, red arrows) [3]. It was also shown that T wave changes in CM show ‘accumulation’ in memory, persisting days or weeks after provoking stimulus, the physiological entity commonly described in neural tissue [3]. Furthermore, CM EKG changes after artificial ventricular pacing are present regardless of the underlying rhythm (supra/idioventricular) and are independent of the heart rate or QRS duration. The problem occurs due to the fact that these T-wave changes can mimic pattern seen in transient, often asymptomatic, proximal left anterior descending artery (LAD) occlusion (Wellens’ syndrome), the entity which has poor prognosis unless urgently revascularized [9,10]. An important difference in T wave axis allows discrimination of CM from ischemic T-wave inversions, regardless of the coronary artery involved. A cornerstone study showed that the combination of a positive T wave in aVL, a positive or isoelectric T wave in lead I, and a maximum T-wave inversion in the precordial leads that was greater than the T-wave inversion in lead III was 92% sensitive and 100% specific for T-wave inversions induced by cardiac memory from right-ventricular pacing with the most important distinguishing feature being difference in leads I and aVL (Figure 1, blue arrows) [11]. The difference exists because ischemia usually involves left ventricle hence ischemic TWI have a rightward axis in the frontal plane while the vast majority of pacemaker leads are implanted in or close to the right ventricular apex producing left superior QRS axis during ventricular pacing with QRS positive in leads I and aVL leading to positive T-waves in those leads during CM [10,11]. Although never conﬁrmed with larger prospective trials, these criteria have been used successfully in clinical practice as evident in all available research following this study.

In our case, although immediately recognized as possible CM changes on initial EKG, and despite the 100% specificity that these changes indicate (as discussed above), initial constellation of his numerous risk factors, symptoms and new TWI seen on EKG were bothersome for severe coronary ischemia. Although this syndrome, if acute, would require immediate cardiac catheterization, given the recognition of CM and possible other explanations for patient’s symptoms (lung disease, psychiatric disease), we were prompted to obtain a nuclear stress test first. When the nuclear stress test indicated ischemia, our next step was cardiac catheterization which showed severe two vessel disease as described above. It is interesting that at this point, specificity of CM EKG characteristics were put to test, due to physical proof of coronary artery disease and EKG resolution of TWI after the procedure (Figure 4). The night after the procedure, our patient had pacing activity seen on telemetry monitor and the EKG obtained afterwards showed again the typical changes characteristic for CM (Figure 5, blue arrows), confirming that initial EKG changes were not due to ischemia.

Recently finished “Ischemia Trial”, failed to show that routine invasive therapy was associated with a reduction in major adverse ischemic events compared with optimal medical therapy among patients with stable ischemic heart disease and moderate to severe myocardial ischemia on noninvasive stress testing [12-14]. Our patient would fit all the inclusion criteria and would not meet any of the exclusion criteria of this trial. Based on the study design of Ischemia Trial, however, after we recognized that our patient’s EKG changes demonstrated CM and not acute ischemia, and after the positive non-invasive stress tests, he should have undergone a CT angiography to exclude severe left main coronary artery disease, and if so, should continue with medical therapy and lifestyle changes alone. It is not specified if patients with pacemakers underwent the same protocol, since device leads can obscure the obtain images, although these problems could be solved using some advanced CT techniques [15-17]. Since we needed some modality to evaluate coronary anatomy, our patient instead underwent coronary angiography which did not show left main disease. At this point, even though it does not follow the exact pathway as in the study, question emerges - was it the right step to abort the stent placement. It is hard for a physician to restrain from the urge to do something to help a patient, especially when widening a narrowed artery is just one move away. As we learned from our case and as it is shown in the reported trial, less is sometimes more, and in selected patients, non-invasive, medical strategy is the right path. One thing remains, EKG criteria for cardiac memory once again got 'validated' in this very complicated patient, and physicians should take into account these EKG finesses that can change the whole diagnostic and therapeutic pathway and most importantly, change the outcome the patient will have.

## Conclusions

It remains a dilemma about the right course of action in high ischemic risk patients with EKG changes of CM. Our patient had multiple warning signs indicating cardiovascular ischemic cause of his chest pressure. Even though alternative diagnoses including CM were offered initially, the significance of ischemic etiology could not be put aside. Although retrospective studies demonstrated 100% specificity of the syndrome, confirmed by individual studies and case reports (including ours), larger prospective trials are still needed. Until then, as we demonstrated in our case, CM is still a diagnosis of exclusion and an interesting electrocardiographic curiosity that will continue to bring more challenges in everyday clinical practice.
